# Soil Nitrate Nitrogen Content and Grain Yields of Organically Grown Cereals as Affected by a Strip Tillage and Forage Legume Intercropping

**DOI:** 10.3390/plants10071453

**Published:** 2021-07-15

**Authors:** Aušra Arlauskienė, Viktorija Gecaitė, Monika Toleikienė, Lina Šarūnaitė, Žydrė Kadžiulienė

**Affiliations:** 1 Joniškelis Experimental Station, Lithuanian Research Centre for Agriculture and Forestry, Joniškėlis, LT-39301 Pasvalys, Lithuania; ausra.arlauskiene@lammc.lt (A.A.); viktorija.gecaite@lammc.lt (V.G.); 2 Lithuanian Research Centre for Agriculture and Forestry, Institute of Agriculture, Instituto al. 1, Akademija, LT-58344 Kėdainiai, Lithuania; monika.toleikiene@lammc.lt (M.T.); zydre.kadziuliene@lammc.lt (Ž.K.)

**Keywords:** black medick, Egyptian clover, inorganic nitrogen, mulch, nitrogen yield, white clover

## Abstract

Reducing tillage intensity and increasing crop diversity by including perennial legumes is an agrotechnical practice that strongly affects the soil environment. Strip tillage may be beneficial in the forage legume–cereals intercropping system due to more efficient utilization of biological nitrogen. Field experiments were conducted on a clay loam Cambisol to determine the effect of forage legume–winter wheat strip tillage intercropping on soil nitrate nitrogen (N-NO_3_) content and cereal productivity in various sequences of rotation in organic production systems. Forage legumes (*Medicago lupulina* L., *Trifolium repens* L., *T.* *alexandrinum* L.) grown in pure and forage legume–winter wheat (*Triticum aestivum* L.) strip tillage intercrops were studied. Conventional deep inversion tillage was compared to strip tillage. Nitrogen supply to winter wheat was assessed by the change in soil nitrate nitrogen content (N-NO_3_) and total N accumulation in yield (grain and straw). Conventional tillage was found to significantly increase N-NO_3_ content while cultivating winter wheat after forage legumes in late autumn (0–30 cm layer), after growth resumption in spring (30–60 cm), and in autumn after harvesting (30–60 cm). Soil N-NO_3_ content did not differ significantly between winter wheat strip sown in perennial legumes or oat stubble. Winter wheat grain yields increased with increasing N-NO_3_ content in soil. The grain yield was not significantly different when comparing winter wheat–forage legume strip intercropping (without mulching) to strip sowing in oat stubble. In forage legume–winter wheat strip intercropping, N release from legumes was weak and did not meet wheat nitrogen requirements.

## 1. Introduction

Crop rotation diversification using legumes has been advocated as one of the solutions to improve crop system resilience to multiple environmental stresses and use of nitrogen (N) resources [1,2]. The cultivation of perennial forage legumes in arable organic farming has shown encouraging results [3]. However, their cultivation often is limited by biological and economic factors. In order to increase commercial production, various practices involving intercropping forage legumes with main crops can be applied (as service crops). By mixing several plant species in one field, ecological principles based on biodiversity, plant interactions, and other mechanisms that promote crop self-regulatory functions can be applied in practice [4,5]. The advantages of intercropping include higher overall productivity, better control of pathogens and pests, strengthening of ecological services, and higher profitability of the crop [6]. Much research has been done on intercropping forage legumes with cereals for short periods (a year) [1,7]. When trying to keep forage legumes for a longer period and resowing cereals, farmers face problems related to sowing cereals and managing the competitiveness of plants [8].

Having ploughed in forage legumes or their green mass, the yield of winter wheat grown afterwards increased significantly [9]. However, when forage legume or grass-legume leys are ploughed-in, there is a high risk of nitrate leaching, especially on sandy soils [10,11]. Some studies have shown that skipping autumn tillage, or at least postponing it before sowing spring cereals, can reduce nitrate losses by up to 25% during winter [12]. Other studies have shown slower mineralization of forage legume mass [13] and smaller nitrogen leaching following less intensive tillage [14]. However, the effect varies widely with the crop and soil type and climatic conditions [15]. No-tillage improves soil structure, its biological activity, and nutrient cycling, and it increases soil water retention capacity and efficiency of its use [16,17]. In addition to savings in energy and labor, reduced tillage can also protect soil from erosion or loss of organic matter in the topsoil [18]. Numerous research data show that no-tillage/reduced tillage temporarily reduces yields [19,20,21,22]. This decrease depends on the plant species, hydrothermal conditions, method of plant residue management, and nitrogen fertilizer rates. Plant yields increased only with the long-term application of no-till practices [19,20]. However, some sources claim that the benefits of no-tillage agriculture are highly overstated [23].

The use of reduced tillage in organic farms often causes problems not only in terms of yield reduction but also in terms of weed infestation [24,25] and disturbed nutrient cycling [22,26]. Organic farming relies on a combination of different practices, and a reduction in one area (e.g., tillage) requires intensification in another (e.g., the diversification of the crop rotation) [22]. Long-term no-tillage can improve soil properties and crop yield when combined with multicropping and diverse rotations, cover crops, and manure application [16,19,27,28]. Another very important condition is the permanent covering of the soil with plant residues [28,29]. In low-input systems, mulching enhances soil fertility by maintaining or increasing soil organic carbon stocks [30] and stabilizing the physical properties of the soil [16].

Strip tillage is the form of conservative tillage, in which soil is disturbed only for sowing rows, and the spaces between rows are not tilled. Strip tillage combines the advantages of conventional and no-tillage, creating two soil zones with different properties and functions [31]. Favorable conditions for seed germination and plant growth are created in the sowing zone, and the no-tilled zone [32] serves to restore soil fertility, suppress weeds, and reduce erosion [30]. Combining cover crops and living mulches with strip tillage brings many benefits to crop production. Living mulches are multifunctional components that can be used to address key challenges faced by the arable sector such as erosion control, reduction in surface water pollution, unused soil nitrogen recycling, declining soil productivity, structure and health, and weed control [33]. Future research on intercrops should focus on the development of the relevant crop management practices to avoid yield losses [34,35]. 

We hypothesized that winter cereal–forage legume strip intercropping with living or dead mulch technologies could optimize the mineralization of crop residues and increase productivity in the long term. The objective of the study was to determine the effect of winter wheat (*Triticum aestivum* L.)–black medick (*Medicago lupulina* L.), winter wheat–white clover (*Trifolium repens* L.), and winter wheat–Egyptian clover (T. *alexandrinum* L.) strip tillage intercrop management on the soil nitrate N content and cereal productivity compared to crop sequences without forage legumes and with conventional tillage.

## 2. Materials and Methods

### 2.1. Experimental Site, Soil, and Design

Field experiments were conducted at Joniškėlis Experimental Station of the Lithuanian Research Centre for Agriculture and Forestry (LAMMC) in the northern part of Central Lithuania’s lowland in 2018–2019. The soil at the experimental site was classified as an *Endocalcari-Endohypogleyic Cambisol* (Siltic, Drainic) [36], the texture of which is clay loam on silty clay with deeper lying sandy loam. The topsoil pH (0–25 cm) is close to neutral (6.1), medium in phosphorus (146 mg P_2_O_5_ kg^−1^), high in potassium (276 mg K_2_O kg^−1^), and moderate in humus (2.54%). 

The main crop in 2018 was spring oats (*Avena sativa* L.), cv. ‘Migla DS’ (O); these were undersown with black medick cv. ‘Arka 133 DS’ (O+BM), white clover cv. ‘Nemuniai’ (O+WC) and Egyptian clover cv. ‘Cleopatra’ (O+EC). Oat and forage legumes (BM, WC, and EC) were also grown in monocrops. In 2019, winter wheat cv. ‘Gaja’ was grown in monocrops (WW) and intercropped with forage legumes (BM+WW, WC+WW, EC+WW). Twelve winter wheat management strategies with two soil tillage treatments—conventional deep inversion tillage and strip tillage—were compared. The treatments included pure stands of wheat grown after four monocrops using conventional tillage and sowing (CTS): O–WW(CTS), BM–WW(CTS), WC–WW(CTS), and EC–WW(CTS); and grown after oat monocrop and oat-forage legume intercrop using strip tillage (STS): O–WW(STS), O+BM–BM+WW(STS), O+WC–WC+WW(STS), and O+EC–EC+WW(STS). To reduce the competitiveness of forage legumes in strip intercropping systems (BM+WW, WC+WW), the forage legume was mulched once (M) or twice (2M) (Table 1). The mass of Egyptian clover froze in 2018–2019 winter (dead mulch). The control treatments included winter wheat grown as a sole crop in a cereal sequence: O–WW(CTS). On the 24th of April 2020 spring wheat was sown on all treatments at a density of 450 seeds m^−2^. The experimental plots were designed as a complete one-factor randomized block with four replicates. The size of individual plot was 6 × 20 m.

### 2.2. Experimental Setup 

The oats were sown on 23 April 2018 at a rate of 450 seeds m^−2^ using a narrow spacing drill (with 0.125 m row spacing) at 3 cm depth. The forage legume species were intercropped in oats on 25 April 2018 at a rate of 50 seeds m^−2^. The forage legume seeds were sown at 2 cm depth using a narrow spacing drill. The conventional tillage and sowing methods were used. After harvesting the oats, the straw was chopped and spread. Forage legumes in monocrops were mulched twice during the growing season (mid-July and late summer before ploughing), and those intercropped were mulched once in late summer. In conventional tillage (according to the respective treatments), the following were used: deep inversion tillage and a pre-sowing cultivation unit. The winter wheat was drill-seeded at a rate of 450 seeds m^−2^ with 0.125 m row spacing, at a depth of 2.5–3.0 cm. In the strip tillage system, involving one-pass tillage, winter wheat sowing operations were performed using a Pro Till 3T hybrid machine manufactured by Mzuri (Pershore, UK) (http://mzuri.eu/pro-til/, accessed on 5 July 2021) The row spacing was 0.33 m, the sowing depth was also 2.5–3.0 cm, and it was sown at a density of 380 seeds m^−2^. In October 2019, after growing winter wheat (straw was chopped and spread by the harvesting machine), each plot was ploughed. Grain yield was harvested when the majority of crops had reached hard dough stage (BBCH 87). Each experimental plot was harvested with a small plot combine harvester. The experimental sites were managed with no use of fertilizers and pesticides. 

### 2.3. Weather Conditions 

**Figure 1:**
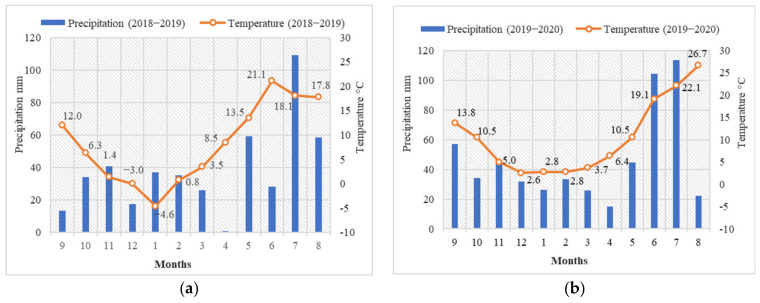
Monthly mean temperature and precipitation at the experimental sites during the periods 2018–2019 (**a**) and 2019–2020 (**b**).

The weather data were obtained from the meteorological station, located 0.5 km away from the experimental site (Figure 1). 

Weather conditions varied between years. The fall in 2018 was dry, which resulted in lower mineral N migration to deeper soil layers, whereas in 2019 the fall was warmer with more sufficient rainfall than in 2018. The winter periods also differed. The winter of 2018–2019 was close to the standard climate normal (SCN). December 2019 to February 2020 was characterized by a positive average daily temperature that was not usual in Lithuania. In 2019, the weather conditions were close to the SCN with a dry period in April and early May. This may have adversely affected N release and winter wheat nutrition. In 2020, the vegetation period was characterized by an uneven distribution of precipitation: insufficient in the first and excess in the second half of this period. The mean temperatures in June and July were significantly higher than the SCN.

### 2.4. Plant and Soil Analyses

Oat, winter wheat grain, and straw yields (kg ha^−1^) were determined. The grain yield was converted to standard moisture content (14%), and straw was converted to dry matter (DM). Plant samples were dried and ground using a ZM200 ultra-centrifugal mill (Retch, Haan, Germany) with 1 mm mesh sieves. Grain and straw N content (kg ha^−1^) was determined in the sulphuric acid digestates using the Kjeldahl method with a Kjeltec system 1002 (Foss Tecator, Hoganas, Sweden). To determine nitrate (N-NO_3_, kg ha^−1^) nitrogen content, soil samples were collected four times: in autumn after winter wheat sowing, 21 November 2018 (Assessment 1); in spring before winter wheat growth resumed, 25 March 2019 (Assessment 2); in autumn, when all experimental plots were ploughed, 16 October 2019 (Assessment 3); and before spring wheat sowing, 23 April 2020 (Assessment 4); all were at 0–30 cm and 30–60 cm depths. One soil sample consisted of five drills from each plot. Nitrate nitrogen (N-NO_3_) was determined by the ionometric method. The samples were placed in 1 mol L^−1^ KCl extract, *w*/*v* ratio 1:5.

### 2.5. Statistical Analysis 

The data were statistically processed using one-factor analysis of variance as well as correlation and regression methods. The significance of differences among the treatment means was estimated at the 0.05 probability level. Correlations among cereal grain yield and soil N-NO_3_ were determined. Simple linear regression (SLR) was applied. The statistical analysis was performed using ANOVA version 3.1 software and STAT_ENG version 1.5 from the programme package SELEKCIJA.

## 3. Results

### 3.1. Soil Nitrate Nitrogen Content 

**Figure 2:**
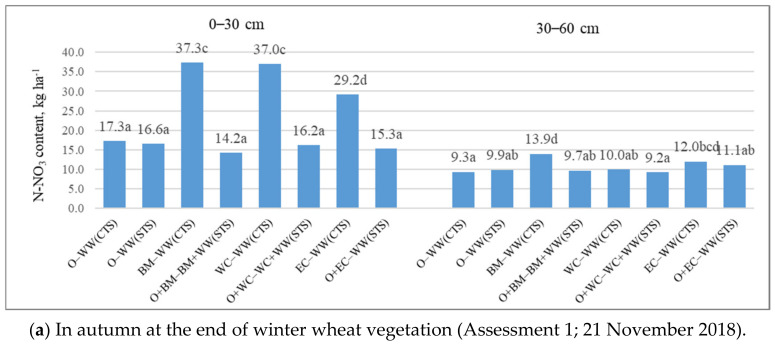

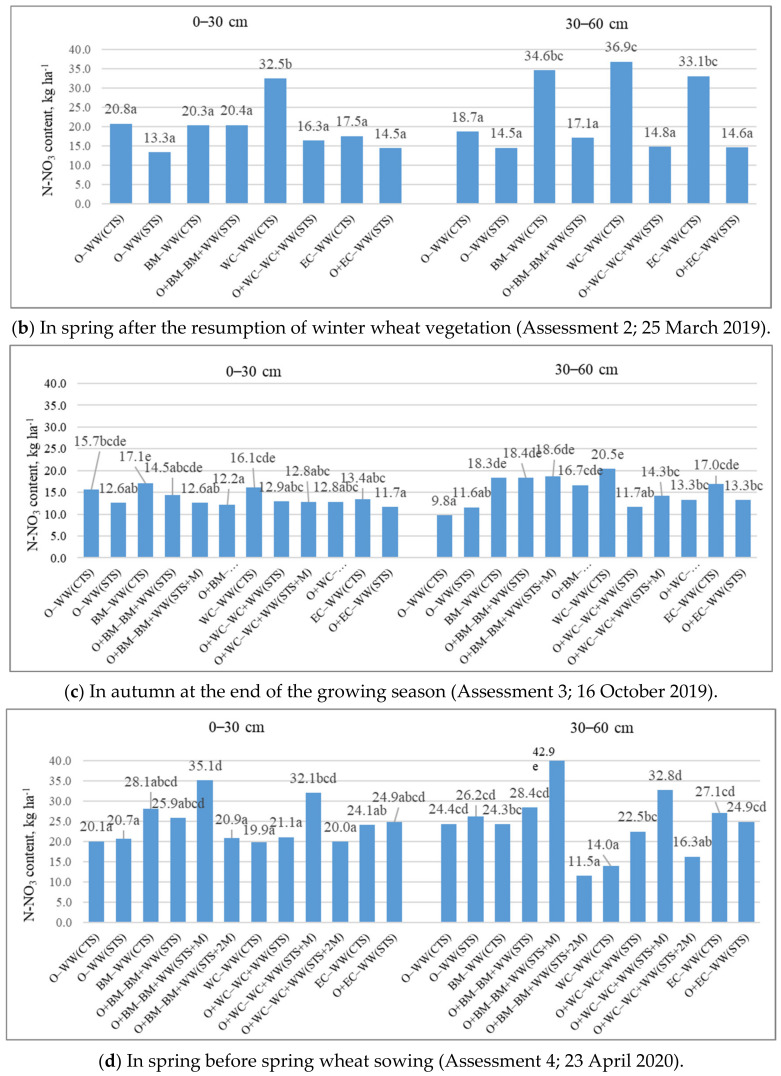
Nitrate nitrogen content at two soil depths on four assessment dates (**a**–**d**) in different tillage crop sequences.

The results showed a significant (at *p* < 0.01) influence of the soil tillage system on the N-NO_3_ content in autumn (Assessment 1, Figure 2a). Having ploughed in BM, WC, and EC prior to winter wheat sowing, N-NO_3_ content in the 0–30 cm soil layer was significantly higher than when CTS and STS had been used after oats. Nitrate N concentration was higher by 2.2 times in winter wheat grown after ploughing in BM, 2.1 times after WC, and 68.8% after EC compared to winter wheat sowing after oats (CTS). Strip tillage tended to reduce N-NO_3_ (4.0–17.9%). As N-NO_3_ increased in the upper soil layer, it tended to increase in the deeper ones as well. 

The results showed that with increasing N-NO_3_ (0–30 cm) in autumn, N-NO_3_ values also increased in the spring (Assessment 2, Figure 2b). The dependences of N-NO_3_ content in spring in the deeper soil layer were stronger (r = 0.89, *p* ≤ 0.01) than in the upper (r = 0.45, *p* ≤ 0.05). High concentrations of N-NO_3_ in the soil on the objects with CTS were also noted in early spring. N-NO_3_ content in the 0–30 cm soil layer increased during wheat cultivation after WC (CTS) by 56.2%, and in the 30–60 cm layer after all forage legumes (CTS) by 77.0–97.3%, compared to sowing after oats (CTS). Strip tillage and forage legume–winter wheat intercropping tended to reduce N-NO_3_ in both soil layers compared to sowing wheat after oats (CTS). 

Winter wheat management strategies: O-WW(CTS)–oat–winter wheat (conventional tillage and sowing); O–WW(STS)–oat–winter wheat (strip tillage and sowing); BM-WW(CTS)–black medick–winter wheat (conventional tillage and sowing); O+BM–BM+WW(STS)–oat undersown with black medick–black medick and winter wheat intercrop (strip tillage and sowing); O+BM–BM+WW(STS+M)–oat undersown with black medick–black medick and winter wheat intercrop (strip tillage and sowing, mulching once); O+BM–BM+WW(STS+2M)–oat undersown with black medick–black medick and winter wheat intercrop (strip tillage and sowing, mulching twice); WC-WW(CTS)–white clover–winter wheat (conventional tillage and sowing); O+WC–WC+WW(STS)–oat undersown with white clover–white clover and winter wheat intercrop (strip tillage and sowing); O+WC–WC+WW(STS+M)–oat undersown with white clover–white clover and winter wheat intercrop (strip tillage and sowing, mulching once); O+WC–WC+WW(STS+2M)–oat undersown with white clover–white clover and winter wheat intercrop (strip tillage and sowing, mulching twice); EC-WW(CTS)–Edyptian clover–winter wheat (conventional tillage and sowing); O+EC–EC+WW(STS)–oat undersown with Edyptian clover–Edyptian clover and winter wheat intercrop (strip tillage and sowing); means followed by the same letters are not significantly different at *p* ≤ 0.05.

At the end of the 2019 growing season and before ploughing (Assessment 3), the highest N-NO_3_ content in the 0–30 cm soil layer was found with winter wheat grown after BM and WC using conventional tillage (Figure 2c). Compared to these treatments, strip tillage significantly reduced N-NO_3_ content in the soil when sowing winter wheat into oat stubble, or mulching EC and BM aboveground mass during the growing season. There was slightly more N-NO_3_ in the 30–60 cm soil layer, and differences between the treatments were more pronounced compared to the upper layer. The lowest N-NO_3_ content was found for winter wheat grown after oats (CTS). Growing forage legumes (CTS) before winter wheat increased the N-NO_3_ content in soil significantly (from 73.5% to 2 times) compared to that with oats as a preceding crop (CTS). Strip tillage increased N-NO_3_ significantly, with the exception of sowing into oat stubble and into WC (without mulching), compared to sowing after oats (CTS). The highest N-NO_3_ content in soil was found when intercropping winter wheat with black medick.

In the spring 2020, significantly higher N-NO_3_ content in the 0–30 soil layer (74.6 and 59.7% respectively) was noted after ploughing black medick–winter wheat and white clover–winter wheat stubble with the mass mulched once (STS+M) compared to control (Figure 2d). In the deeper soil layer, similar trends in N-NO_3_ content remained, only the differences were larger. When evaluating BM treatments, it was found that significantly higher N-NO_3_ content (76.5%) was observed after ploughing black medick–winter wheat stubble with the mass mulched once (STS+M), and a significantly lower content (73.2%) was found when the mass was mulched twice (STS+2M), compared to BM-WW(CTS). Significantly higher N-NO_3_ content was also noted with one mulching of the aboveground mass of white clover (WC+WW(STS+M)) and without its mulching (WC+WW(STS)) in strip tillage system compared to that with WC in the conventional tillage system (WC-WW(CTS)). Our studies also showed higher N-NH_4_ accumulation in soil as influenced by strip tillage (data not shown).

### 3.2. Cereal Productivity

The forage legume intercrop had no significant effect on oat grain yield compared to the yield of oats grown as a monocrop (Table 2). Winter wheat grain yields of different cropping sequences depended on winter wheat establishment strategies (at *p* < 0.01). Ploughing in forage legumes before winter wheat sowing increased grain yield significantly by 30.6–47.4% compared to the winter wheat grown after oats. Winter wheat strip tillage and sowing into oat stubble reduced the grain yield by a substantial 21.2% compared to conventional sowing into ploughed soil. However, the yield of winter wheat sown in BM (STS and STS+M) and EC (STS) stubble was similar to that of winter wheat sown conventionally after oats. When winter wheat was intercropped with forage legumes (without mulching) or sown into oat stubble, the grain yields did not differ significantly. An upward trend in winter wheat grain yield was observed when applying strip sowing in dead EC mulch.

The total N accumulation in winter wheat harvest (grain + straw) differed between treatments (Table 2). The highest N amount in winter wheat yield was accumulated during its cultivation in the forage legumes sequence with conventional tillage. Having ploughed in forage legumes BM, WC, and EC prior to winter wheat sowing, the N content increased by 46.1%, 71.0%, and 53.5%, respectively, compared to the control treatment. The correlations between the N yield of winter wheat and N-NO_3_ content in the 0–60 cm layer in autumn and spring were statistically significant (r = 0.79 and r = 0.73, *p* ≤ 0.01, respectively).

**Figure 3:**
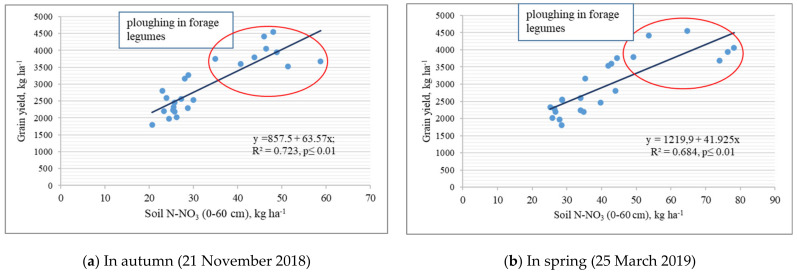
Dependence of winter wheat grain yield on N-NO_3_ content accumulated in soil (0–60 cm) during autumn (**a**) and spring (**b**).

The correlation analysis showed that the yield of winter wheat grain depended on the N-NO_3_ content in the soil (0–60 cm) both in the autumn and spring after the resumption of wheat growth (Figure 3a,b). As the N-NO_3_ content increased from 20.7 to 58.7 kg ha^−1^ (Assessment 1) and from 25.2 to 78.1 kg ha^−1^ (Assessment 2), the yield of winter wheat grain increased two-fold, i.e., from 1804 to 4413 kg ha^−1^. The increase in yield was mainly influenced by ploughing in forage legume mass.

## 4. Discussion

### 4.1. Nitrogen Supply for Winter Wheat

Winter wheat–forage legume intercropping with strip tillage allows retaining forage legumes for a longer period of time owing to the soil enrichment with nitrogen [37]. In the late autumn of 2018, the highest N-NO_3_ content in the 0–30 cm soil layer was with conventional soil tillage, and it was 2.9 times higher than when winter wheat was strip-intercropped with forage legumes. The increase in mineral nitrogen in soil was due to the nitrogen-rich and rapidly decomposing (C:N < 15) mass of forage legumes incorporated during ploughing [9]. Previous studies showed that in the conditions of variable humidity in autumn, most of the mineral nitrogen accumulates in the deeper layer (30–60 cm) after ploughing in forage legume mass [9], and usually this nitrogen is not available, as the main cereal roots have not yet formed [38]. Therefore, during such a period, there is a risk of nitrogen migration into deeper soil layers and its leaching [10]. Based on recent studies, Skaalsveen et al. [15] stated that no-tillage does not reduce nitrogen leaching unless cover crops are grown. Walmsley et al. [39] noted that non-inversion tillage with a cover crop can increase the leaching of dissolved, organically bound nitrogen.

After the resumption of winter wheat growth in the spring of 2019, N-NO_3_ content was analogous to that measured in the autumn, only most of it was in the deeper soil layer (30–60 cm). The application of strip tillage to forage legumes–winter wheat resulted in an average of 43.9% lower N-NO_3_ content in both soil layers combined (0–60 cm) compared to conventional sowing after forage legume ploughing in. This confirms the statement that mineralization intensity and nitrogen supply can be managed by choosing the right tillage method [22,40]. Low soil nitrogen content was determined by several factors. When tilling only the sowing lines, a small mass of forage legumes was incorporated. In addition, some portion of the chopped oat straw (C:N = 80–100) also entered the soil, and the microorganisms may use soil nitrogen to decompose it. Winter wheat and forage legumes, as well as other plants and soil microorganisms, compete for soil nitrogen. Applying no-tillage or reduced tillage methods increases soil density, and reduces aeration and water supply [41], resulting in the slow mineralization of plant residues.

Winter wheat had access to high amounts of nitrogen after ploughing-in forage legumes. Nitrogen supply was reduced by strip tillage. Other researchers also noted that nitrogen supply for the crops can be limited by reduced tillage [20,22]. Plant nutrition is reported to improve by increasing soil biological activity due to shallow soil loosening and incorporation of plant residues [27,42]. Soil moisture and heat retention also improved due to covering the soil surface with plant residues [16]. In our studies, loosening of the sowing strips (13 cm) at a depth of 13–15 cm did not have any more pronounced effect on N-NO_3_ content in spring than sowing into oat stubble. Forage legumes were usually uprooted and laid between rows. Uprooted BM and EC performed the function of dead mulch, and WC took root (as living mulch) in spring and competed with the wheat. Mulching of the aboveground mass of forage legumes and the application of organic fertilizers [43] can reduce competitiveness and increase nitrogen application [24,44]. Nitrogen release from plant mass depends on tillage intensity and mulch mass [26], forage legume density, root morphology, and plant residue quality [45], as well as mulch C:N [22]. Due to the narrow C:N ratio, more nitrogen releases from the shoots (63.4–70.0% of the initial N) and more rapidly than from the roots (27.3–50.7%) [46].

Effective nitrogen utilization depends on the synchronism of its release from plant residues and the requirements of crops. By reducing tillage intensity, nitrogen is released at later stages of crop growth during the growing season [21,47]. In addition, the development and depth of penetration of the main crop roots is also determined by tillage [41]. Our studies (Assessment 3) showed that in most cases higher N-NO_3_ levels remained after ploughing-in forage legumes. However, an increase in N-NO_3_ was observed in the deeper soil layer of the winter wheat–forage legume intercrop compared to the wheat monocrop (CTS). After ploughing-in the intercrop and monocrop in spring, the highest N-NO_3_ content was found in the plots with winter wheat–white clover and winter wheat–black medick intercrops with mulching of forage legume mass on the soil surface (STS+M). 

Other studies have shown that the combination of reduced tillage and organic manure increases the sequestration of organic carbon in the upper soil layer. Using legume in combination with grass increases total soil organic C and N content and reduces negative environment impacts [5]. The addition of plant residues from green manure and their incorporation into soil activates the subsequent physico-chemical protection of organic carbon [48]. Prolonged application of no-tillage and the accumulation of organic matter of different availability in the soil increases the soil’s capacity to supply available nitrogen by generating intrinsic resources [49]. This contributes to an improvement in plant nutrition with nitrogen in the long run. However, the effect varies largely with the crop, soil type, and climatic conditions.

### 4.2. Variation in Winter Wheat Yield

Winter wheat is moderately sensitive to no-tillage [19]. Our results show that the grain yield of winter wheat decreased when applying strip tillage in oat stubble by 21.2%, in BM by 14.1% and in WC by 23.4% (as a living mulch), and in EC by 8.9% (as a dead mulch) compared to conventional tillage after oats. In other studies, the crop yields under conventional tillage were 34% higher compared to those under strip tillage [20]. Some studies found that strip tillage in organic farming decreased yields due to inadequate weed control and decreased nutrient availability [20,21]. When wheat is intercropped with clover (as a living mulch), wheat yields are mostly reduced due to plant competition [35] for light, moisture, and nitrogen, and crop losses can reach 10–25% compared to the wheat monocrop [47]. The suitability of plants for intercropping depends on differences in the root system of forage legumes and the main crop, the rate of regrowth of forage legumes, uniformity of soil cover, moisture demand, and competitive properties [22,50]. When switching from conventional tillage to no-tillage, crop yields tend to decline. This decline can last 5 years or more [19]. This is often related to soil carbon sequestration and the stabilization of beneficial soil properties [51,52]. Application of no-tillage improves soil health and sometimes can increase crop yields [28]. Covering the soil surface with plant residues, increasing plant diversity (especially perennial grass cultivation), and using mulch are essential no-tillage conditions [22,42]. In our studies, when the plant residue mass was increased using dead EC mulch, the yield of winter wheat grain was 15.7% higher compared to sowing into oat stubble. The results showed that the use of living mulch led to competition between WC and winter wheat. Suppression of forage legumes in spring is required to reduce competition between plants [37]. In our studies, mulching did not significantly increase the yield of winter wheat. Recent studies suggest that in order to improve the efficiency of plant resource use, optimizing planting geometry for cereal–forage legume intercropping systems is required [53]. Cooper et al. [21] stated that using inversion tillage to a shallow depth is the most suitable for organic farming as it reduces yields (5.5%) marginally, but it significantly increases soil carbon stocks and controls the spread of weeds better. According to Casagrande et al. [8], the most important motivation for reduced tillage is the preservation of soil fertility, with the challenges being related to crop management, machinery, and fertility.

## 5. Conclusions

Ploughing in forage legume monocrops for winter wheat increased N-NO_3_ content after sowing wheat in late autumn, after growth had resumed in spring and after harvesting (mostly in the 30–60 cm soil layer). Strip tillage and winter wheat–forage legume intercropping reduced N-NO_3_ content by an average of 43.9% in spring compared to conventional tillage and sowing after the forage legume monocrop. An increase in N-NO_3_ in the soil of forage legume–winter wheat intercrops was observed only after harvesting winter wheat in late autumn compared to the wheat monocrop (CTS). After ploughing in intercrops and monocrops, the highest N-NO_3_ content in spring 2020 was noted in winter wheat–white clover and winter wheat–black medick intercrops with mulching of forage legume mass on the soil surface (STS+M).

Our results showed that grain yields decreased by 21.2% with strip tillage and winter wheat sowing into oat stubble compared to conventional tillage and sowing after oats. The grain yield from strip tillage was the highest after sowing winter wheat in dead EC mulch. The application of winter wheat intercropping with BM and WC reduced the yield of winter wheat grain by 14.1% and 23.4%, respectively, compared to conventional tillage and sowing after oats. This indicates greater competition from WC compared to BM. Mulching of the aboveground mass of forage legumes did not significantly increase the yield of winter wheat grain. Future research should focus on improving forage legume–cereal intercrop management in order to optimize plant residue mineralization and to obtain satisfactory and stabile cereal yields.

## Figures and Tables

**Table 1:** Crops, tillage, and sowing methods.

Rotation Sequences					Treatments
2018		2019			
Crops	Soil Tillage and WW Sowing	Crops	Mulching of Forage Legumes	Soil Tillage in Autumn	
Oat (Control)	Conven- tional tillage: mass of forage legumes and oat straw incorporated into the soil during ploughing at 23–25 cm depth (26 September 2018) and sowing (27 September 2018)	Winter wheat (WW)		Conventional tillage: cereal straw incorporated into the soil during ploughing at 23–25 cm depth (25 October 2019)	O–WW(CTS)
Black medick (BM)					BM–WW(CTS)
White clover (WC)					WC–WW(CTS)
Egyptian clover (EC)					EC–WW(CTS)
Oat	Strip tillage and sowing (26 September 2018)			Conventional tillage: mass of forage legumes and cereal straw incorporated into the soil during ploughing at 23–25 cm depth (25 October 2019 )	O–WW(STS)
Oat and undersown black medick (O+BM)		Black medick–winter wheat intercrop (BM+WW)	No		O+BM–BM+WW (STS)
			once (M) (31 May 2019)		O+BM–BM+WW (STS+M)
			twice (2M) (26 June 2019)		O+BM–BM+WW (STS+2M)
Oat and undersown white clover (O+WC)		White clover–winter wheat intercrop (WC+WW)	No		O+WC–WC+WW (STS)
			once (M) (31 May 2019)		O+WC–WC+WW (STS+M)
			twice (2M) (26 June 2019)		O+WC–WC+WW (STS+2M)
Oat and undersown Egyptian clover (O+EC)		Egyptian clover–winter wheat intercrop (EC+WW)	Dead mulch		O+EC–EC+WW (STS)

**Table 2:** Cereal yields in different cropping sequences using conventional and strip tillage.

2018		2019		
Crops	Grain Yield of Oat kg ha^−1^	Crops, Tillage and Sowing Methods	Grain Yield of Winter Wheat kg ha^−1^	Nitrogen Accumulation kg Nha^−1^
O	3360 a	WW (CTS)	2808 d	57.9 c
O	3360 a	WW (STS)	2212 abc	47.1 abc
BM	-	WW (CTS)	3668 e	84.6 d
O+BM	3372 a	BM+WW (STS)	2412 bcd	50.5 abc
O+BM	3372 a	BM+WW (STS+M)	2449 bcd	50.2 abc
O+BM	3372 a	BM+WW (STS+2M)	2340 abc	46.7 abc
WC	-	WW (CTS)	4138 g	99.0 f
O+WC	3409 a	WC+WW (STS)	2151 ab	44.5 ab
O+WC	3409 a	WC+WW (STS+M)	1992 a	41.0 a
O+WC	3409 a	WC+WW (STS+2M)	2485 bcd	50.3 abc
EC	-	WW (CTS)	3969 efg	88.9 def
O+EC	3556 ab	EC+WW (STS)	2559 cd	53.9 bc

Note: O—oat, BM—black medick, WC—white clover, EC—Egyptian clover, O+BM—oat undersown with black medick, O+WC—oat undersown with white clover, O+EC—oat undersown with Egyptian clover; WW(CTS)—winter wheat (conventional tillage and sowing), WW(STS)—winter wheat (strip tillage and sowing), BM+WW(STS)—black medick and winter wheat intercrop (strip tillage and sowing), BM+WW(STS+M)—black medick and winter wheat intercrop (strip tillage and sowing, mulching once), BM+WW(STS+2M)—black medick and winter wheat intercrop (strip tillage and sowing, mulching twice), WC+WW (STS)—white clover and winter wheat intercrop (strip tillage and sowing), WC+WW (STS+M)—white clover and winter wheat intercrop (strip tillage and sowing, mulching once), WC+WW (STS+2M)—white clover and winter wheat intercrop (strip tillage and sowing, mulching twice), EC+WW (STS)—Egyptian clover and winter wheat intercrop (strip tillage and sowing); means followed by the same letters are not significantly different at *p* ≤ 0.05.

## Data Availability

The datasets used and/or analyzed during the current study are available from the corresponding author on reasonable request.
